# Evolution of the Arsenal of Legionella pneumophila Effectors To Modulate Protist Hosts

**DOI:** 10.1128/mBio.01313-18

**Published:** 2018-10-09

**Authors:** Ashley Best, Yousef Abu Kwaik

**Affiliations:** a Department of Microbiology and Immunology, School of Medicine, University of Louisville, Louisville, Kentucky, USA; b Center for Predictive Medicine, University of Louisville, Louisville, Kentucky, USA; University of Texas Health Science Center at Houston

## Abstract

Within the human host, Legionella pneumophila replicates within alveolar macrophages, leading to pneumonia. However, L. pneumophila is an aquatic generalist pathogen that replicates within a wide variety of protist hosts, including amoebozoa, percolozoa, and ciliophora. The intracellular lifestyles of L. pneumophila within the two evolutionarily distant hosts macrophages and protists are remarkably similar. Coevolution with numerous protist hosts has shaped plasticity of the genome of L. pneumophila, which harbors numerous proteins encoded by genes acquired from primitive eukaryotic hosts through interkingdom horizontal gene transfer. The Dot/Icm type IVb translocation system translocates ∼6,000 effectors among Legionella species and >320 effector proteins in L. pneumophila into host cells to modulate a plethora of cellular processes to create proliferative niches. Since many of the effectors have likely evolved to modulate cellular processes of primitive eukaryotic hosts, it is not surprising that most of the effectors do not contribute to intracellular growth within human macrophages. Some of the effectors may modulate highly conserved eukaryotic processes, while others may target protist-specific processes that are absent in mammals. The lack of studies to determine the role of the effectors in adaptation of L. pneumophila to various protists has hampered the progress to determine the function of most of these effectors, which are routinely studied in mouse or human macrophages. Since many protists restrict L. pneumophila, utilization of such hosts can also be instrumental in deciphering the mechanisms of failure of L. pneumophila to overcome restriction of certain protist hosts. Here, we review the interaction of L. pneumophila with its permissive and restrictive protist environmental hosts and outline the accomplishments as well as gaps in our knowledge of L. pneumophila-protist host interaction and L. pneumophila’s evolution to become a human pathogen.

## Abstract (precis)

Within the human host, Legionella pneumophila replicates within alveolar macrophages, leading to pneumonia. However, L. pneumophila is an aquatic generalist pathogen that replicates within a wide variety of protist hosts, including amoebozoa, percolozoa, and ciliophora.

## LEGIONELLA PNEUMOPHILA IS AN ENVIRONMENTAL GENERALIST PARASITE OF PROTISTS

Legionella pneumophila has intrigued scientists since it first appeared on the world stage in 1976 and continues to do so today. L. pneumophila is a Gram-negative facultative intracellular bacterium that proliferates within alveolar macrophages, causing Legionnaires’ disease (1). It was first suggested by Rowbotham, in 1980, that Legionella could live intracellularly within amoebae, specifically Acanthamoeba and Naegleria (2). Legionella has adapted to and coevolved with numerous protist species in the environment (3, 4) and is mostly part of biofilms (5–9). Of the 8 phyla under Protozoa, only Amoebozoa (17 species) and Percolozoa (7 species) have been shown to harbor L. pneumophila (Table 1). Ciliates like Tetrahymena spp., Paramecium spp., Oxytricha bifaria, and Stylonychia mytilus (10–12), which are hosts for L. pneumophila (Table 1), are no longer considered to be part of the kingdom Protozoa but are of the kingdom Chromista (also known as Chromalveolata), introduced under the modern taxonomy of the Cavalier-Smith system (13). This reclassification gives insight into the wide diversity and broad range of unicellular environmental hosts for Legionella as a generalist pathogen.

**TABLE 1:** Protist species that can support intracellular growth of *Legionella pneumophila*

Protozoan species	Phylum^*a*^	Reference(s)
Acanthamoeba castellanii, A. culbertsoni, A. hatchetti, A. polyphaga, A. royreba, A. astronyxis, A. jacobsi, A. palestinensis, A. lenticulata	Amoebozoa	2, 132, 172–177
Balamuthia mandrillaris	Amoebozoa	178
Cochliopodium minus	Amoebozoa	179
Comandonia operculata	Amoebozoa	174
Dictyostelium discoideum	Amoebozoa	16, 17
Echinamoeba exundans	Amoebozoa	171, 173
Filamoeba nolandi	Amoebozoa	174
Hartmannella cantabrigiensis	Amoebozoa	53, 173, 174
Vermamoeba vermiformis (previously, Hartmannella vermiformis)	Amoebozoa	53, 67, 173, 174
Naegleria lovaniensis, N. fowleri, N. gruberi, N. jadini	Percolozoa	2, 29, 172, 180
Vahlkampfia jugosa (Tetramitus jugosa), V. ustiana	Percolozoa	53, 174, 181
Willaertia magna	Percolozoa	182
Oxytricha bifaria	Ciliophora	10
Tetrahymena tropicalis, T. pyriformis, T. thermophila, T. vorax	Ciliophora	11, 183, 184
Stylonychia mytilus	Ciliophora	10
Paramecium caudatum, P. tetraurelia	Ciliophora	12, 185

a All phyla are of the kingdom Protozoa, except for Ciliophora, which is of the kingdom Chromista.

Interestingly, although protists graze on bacteria and digest them as a food source, Legionella spp. have been shown to be the most adapted to coopt protist digestion. Legionella hijacks the protist host as an intracellular proliferation niche in the aquatic environment and remains the most prolific human pathogen to replicate within various unicellular eukaryotic hosts (14). One of the most commonly studied protist hosts of L. pneumophila is Dictyostelium discoideum, a social amoeba within the phylum Amoebozoa (4, 15–20). D. discoideum is not a common natural host of L. pneumophila but has the benefit of being a well-described genetically amenable model organism that is permissive to L. pneumophila infection (15–17, 19, 21).

Exploration of the ability of L. pneumophila to replicate intracellularly within other phyla of Protozoa or Chromista could possibly elucidate an even greater host range. As a place to start, Euglenozoa and Choanozoa have been identified in biofilms that contained L. pneumophila, indicating the potential to interact with L. pneumophila (22). Rhinosporidum spp. are members of the Choanozoan phylum, have been shown to be associated with Legionella-containing biofilms, and are considered a possible host (22). Rhinosporidium seeberi is a human parasite that infects the mucosa of the nasal cavity, causing the development of a mass-like lesion, and is primarily found in tropical areas around Sri Lanka and India (23). L. pneumophila can also survive extracellularly in the environment within biofilms (9, 24). These biofilms usually exist with other microbial communities, which could provide L. pneumophila with the nutrients they require to support growth (25). However, the relationship between L. pneumophila and other members of the biofilm communities is poorly understood. One of the limiting factors in studying these alternative protist hosts of L. pneumophila is the limited genomic availability of protists (26). Understanding of the genomic architecture of potential new hosts would contribute greatly to our understanding of coevolution of L. pneumophila with various protist hosts.

It would be valuable to determine if L. pneumophila is capable of infecting any other phyla of Protozoa (Choanozoa, Euglenozoa, Loukozoa, Metamonada, Microsporidia, and Sulcozoa) or Chromista (13, 27). Many members of the euglenozoan phyla possess chloroplasts and/or lack a classical mitochondrion (28). They are most closely related to Percolozoa, which L. pneumophila is capable of infecting (13). During intracellular infection, the mitochondria of the host cell have been shown to be closely associated with the Legionella*-*containing vacuole (LCV) (29–31). To further understand the importance of this close association, these organisms are a potential candidate for future study. Some examples of possible areas of inquiry are as follows. Would chloroplasts be found in close proximity to the LCV? Does L. pneumophila harbor specific proteins that interact with chloroplasts? Alternatively, members of the Metamonada phyla of Protozoa lost their mitochondria but still retain mitochondrial relics like mitosomes and hydrogenosomes (32). What role would these structures have on intracellular replication of Legionella, if the bacteria can even replicate intracellularly within these organisms?

## NUTRITIONAL ADAPTATION AND COEVOLUTION OF L. PNEUMOPHILA WITH PROTISTS

Protists in the environment serve as the source of carbon and energy, since Legionella cells are nutritionally dependent on the host’s amino acids (33). Legionella’s unique nutrient requirements are representative of an intracellular lifestyle, and thus, the bacteria are not commonly found growing free in the environment (34). Amino acids, particularly serine and cysteine, are used to generate pyruvate to feed into the tricarboxylic acid (TCA) cycle, which is the main metabolic pathway in L. pneumophila for generation of energy (25, 33, 35–37). Glucose is minimally used through glycolysis, but metabolized mainly through the Enter-Doudoroff pathway (35, 38, 39). Protists obtain their nutrients from consuming bacteria, yet legionellae have evolved to evade the host’s attempts at consuming them, a trait that is not unique to legionellae: Mycobacterium sp., Francisella tularensis, Cryptococcus neoformans, and others, have transient associations with amoebae (40–42). Yet, no other microbe has been shown to be a generalist pathogen with such a broad host range of unicellular eukaryotes as Legionella, and no other pathogen replicates within protists as well as L. pneumophila.

**FIG 1:**
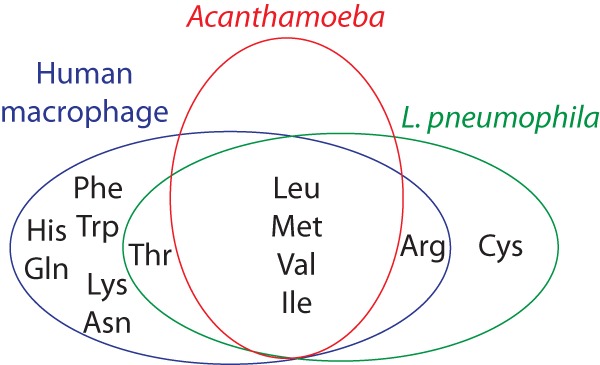
Amino acid auxotrophy in human macrophages, Acanthamoeba, and L. pneumophila. There is considerable overlap in auxotrophy between L. pneumophila and its most common environmental host, Acanthamoeba. Many of these auxotrophies are also seen in human macrophages, the accidental host.

**FIG 2:**
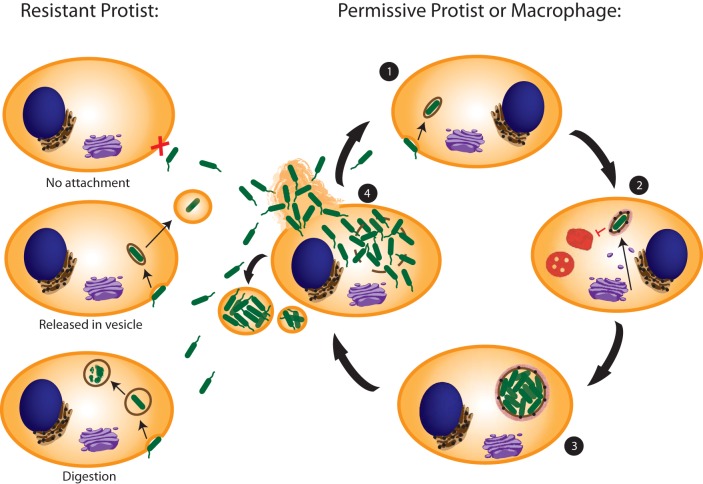
Interactions of L. pneumophila with protist and macrophage host cells. Resistant protist hosts prevent intracellular replication of L. pneumophila through three mechanisms: preventing attachment, releasing L. pneumophila in a vesicle, and digestion. Intracellular replication can be successful if L. pneumophila can attach and enter the host (step 1), where it can then establish the LCV by modifying the vacuole with ER-to-Golgi complex-derived vesicles and prevent lysosome fusion (step 2). Within the replicative LCV, the bacteria replicate in high numbers (step 3). After many rounds of replication, the bacteria break out of the LCV into the cytosol, undergo a couple of rounds of replication, and transition into the transmission stage, becoming flagellated to aid in egress from the host and finding the next host (step 4). The cycle is then repeated if the bacterium encounters another permissive host, which could be a human macrophage.

Because amino acids, particularly serine, are the preferred carbon and energy source, life within the amoebae may have become preferable due to coevolution and ease of access to amino acids of protists (33, 43). L. pneumophila is auxotrophic for seven amino acids (cysteine, leucine, methionine, valine, threonine, isoleucine, and arginine) (Fig. 1) (35, 44, 45). These auxotrophies are synced with their environmental hosts, indicating nutritional coevolution and adaptation to the protist hosts (Fig. 1) (34, 46, 47). Acanthamoeba, one of the most prevalent environmental hosts for Legionella, is auxotrophic for arginine, isoleucine, leucine, methionine, and valine (Fig. 1) (48). Humans are auxotrophic for histidine, isoleucine, leucine, lysine, methionine, phenylalanine, threonine, tryptophan, and valine (49). Macrophages are additionally auxotrophic for glutamine and asparagine (Fig. 2) (49). Given that the macrophage cannot generate 12 amino acids through *de novo* synthesis, and thus has to rely on uptake from the environment, it may represent a nutrient-limiting, energy-deficient host compared to intracellular replication within protists (49). It is unknown if this limitation does result in less robust replication. Simple studies looking at supplementation of the wild-type (WT) L. pneumophila strain with single or multiple amino acids during infection of human macrophages could yield an answer.

The synchronization of auxotrophies with the protist host may allow L. pneumophila to survive through nutrient stresses. Some protists differentiate into the cyst form when encountering environmental stress or as part of their natural life cycle (50). Interestingly, in response to nutrient limitation, L. pneumophila differentiates into a dormant state, and when conditions become more favorable, it becomes metabolically active again within the host (51). This dormant state is classified as “viable but nonculturable” (VBNC) (51, 52).

Entering a VNBC state within an encysted protist may allow L. pneumophila to survive through the same environmental stresses that the protist encounters while ceasing bacterial replication (53–55). Escaping the host, before encystation, and finding a new host with more favorable conditions may provide a replicative advantage, as it has been suggested that encystation is the main process by which amoebae resist L. pneumophila infection (56). However, if the environment into which the bacterium would escape is hostile, it would be a disadvantage to leave the protection of the encysted protist. Given the amount of control L. pneumophila exerts over the fate of its protist hosts, it would not be surprising to find L. pneumophila factors that specifically govern protist-specific cellular processes that are absent in higher eukaryotes.

Some species of Legionella are so dependent on the amoebal host that they cannot be cultured *in vitro* by any means, except by cocultivation with amoebae (51). These organisms are called Legionella-like amoebal pathogens (LLAPs) (57). It may be that LLAPs are nutritionally dependent on their protist host. One LLAP was isolated from a pneumonia patient’s sputum, indicating that LLAPs are capable of causing disease in humans (57). Studying gene loss/gain between LLAPs and L. pneumophila would serve as a means to elucidate the transition from obligate to facultative intracellular pathogen and vice versa.

## THE INTRACELLULAR LIFESTYLE WITHIN PROTISTS AND MACROPHAGES

The largest impact the protist hosts have on human disease is the priming of L. pneumophila for subsequent infection. Amoebae have been referred to as the “Trojan horses of the microbial world” or the “training grounds” for L. pneumophila (14, 58). This is because as legionellae prepare to exit the protist host, they enter a transmissive state, becoming more virulent (14, 25). L. pneumophila cells that have escaped the environmental host are more infectious and can cause a more robust disease in humans (59–61). Protists are also capable of releasing vesicles of respirable size that contain many L. pneumophila cells, thus increasing the dose of bacteria to the individual (Fig. 2) (62).

Whether it is its natural protist host or its accidental host cell (e.g., a human macrophage), both the entry as well as the intracellular life cycle of L. pneumophila are remarkably very similar. In step 1 of Fig. 2, flagellated L. pneumophila attaches the host cell. Attachment of L. pneumophila is host cell specific: the Gal/GalNAc lectin of Vermamoeba vermiformis (previously classified as Hartmennella vermiformis) is used for L. pneumophila attachment, and the mannose binding lection (MBL) is used for attachment to Acanthamoeba castellanii, while complement receptors 1 and 3 are used for human monocytes in a microfilament-dependent manner (63–68). Pili aid in the attachment to human macrophages and Acanthamoeba polyphaga, independent of host factors, and are likely to be involved in attachment to other hosts (69). Immediately upon attachment, L. pneumophila begins to alter the host by translocating protein effectors into the cytosol via the type IVb Dot/Icm translocation system (T4SS), which translocates >320 effector proteins into the host (70–75). Phagocytosis occurs via conventional mechanisms, although a unique form of entry has been observed, called coiling phagocytosis (76, 77).

Within the host, as seen in step 2 of Fig. 2, the bacterium resides within the LCV. To create this protective and permissive niche, L. pneumophila avoids vacuolar acidification and the endosomal-lysosomal degradation pathway (31, 78). The vacuole is rapidly remodeled by intercepting endoplasmic reticulum (ER)-to-Golgi vesicles (74, 79–82). Modification of the vacuole occurs immediately upon uptake (74). This modified vacuole rapidly becomes tubular ER derived (31, 81, 83–85). Additionally, polyubiquitinated proteins rapidly decorate the LCV through the AnkB effector (86) but are counteracted by the RavZ effector (87) and are degraded by the host proteasome as the main source of carbon and energy for L. pneumophila (Fig. 2) (88–92). The types of Legionella metabolism within both hosts are also very similar (93).

L. pneumophila replicates to high numbers within the LCV, with a generation time of ∼1 h, step 3 of Fig. 2. Eventually, by ∼16 h, the bacteria break out from the LCV into the host cytosol, step 4 of Fig. 2 (14, 94–96). The bacteria undergo a few more rounds of replication in the cytosol (94). At this point, nutrient levels in the cytosol are very low, triggering the bacterial alarmone ppGpp and inducing a transition from the intracellular, replicative phase into the virulent transmissive phase (25, 34, 97–100). The intracellular life cycles of L. pneumophila are similar in both protists and human macrophages.

One of the key changes in the transmissive phase is the production of the flagellum, which helps the bacteria to find a new host (25, 100). Free, flagellated bacteria can go on to repeat the cycle within a new host cell (25). It is at this point that infection of humans can occur by aerosolization of infectious particles of free bacteria, bacteria within released vesicles, or even bacterium-filled protists (34, 101). Inhaled bacteria enter the lungs, are taken up by resident alveolar macrophages, and continue the cycle in the same manner as they would in a protist host (31, 102).

## TRANSLOCATION OF AN ARSENAL OF EFFECTORS CONTRIBUTES TO THE BROAD HOST RANGE OF L. PNEUMOPHILA

Successful infection of any host cell by L. pneumophila depends on a functional Dot/Icm T4SS (103–105). Protein substrates translocated by the Dot/Icm T4SS are collectively referred to as “effectors,” which have been shown to modulate a plethora of cellular processes in protists and human macrophages. Within the genus Legionella, an astonishing ∼6,000 effector proteins have been identified (106). Various screens and bioinformatics approaches in L. pneumophila have led to the identification of over 320 effectors translocated by the Dot/Icm T4SS, representing ∼10% of the genome (∼3,200 proteins) (72, 107–109). The translocation of more than 320 effectors into the host cell by L. pneumophila is substantially greater than the next highest number of injected effectors by a pathogen, at >100 by Coxiella burnetii, which is a close relative of L. pneumophila (110). Delivery of a subset of effectors occurs immediately upon attachment and occurs throughout intracellular growth (70, 71).

Intracellularly, the Dot/Icm T4SS machinery is located at the poles of the bacterium (111). Despite the potential to translocate a large number of different effectors, on average, only ∼4 Dot/Icm T4SS translocation structures are located at a pole (111, 112). Surprisingly, nonpolar localization of the Dot/Icm structures results in failure of the pathogen to evade the lysosomes, despite translocating effectors (111). This replication defect suggests localization of effectors at the pole may be required for successful biogenesis of the LCV or effective translocation.

## ARSENAL REDUNDANCY OF L. PNEUMOPHILA EFFECTORS

While single deletion of most effectors of L. pneumophila does not result in a phenotypic defect of intracellular replication, few effector null mutants of L. pneumophila exhibit intracellular growth defects in human or mouse macrophages; this is thought to be due to a functional redundancy of many effectors (73, 74, 113, 114). Even minimizing the L. pneumophila genome by eliminating 31% of the known effectors barely caused any intracellular growth defect in mouse macrophages (114).

Redundancy among the L. pneumophila effectors occurs in different manners: molecular, target, pathway, cellular process, and system redundancies (113). Those redundancies have all been shown in mammalian macrophages. Whether or not these redundancies occur in protist hosts is unknown. As an example of molecular redundancy, members of the SidE family of effectors have been shown to perform the same function on the same host cell target (73). SidE, SdeA, SdeB, and SdeC catalyze the ubiquitination of the host proteins reticulon 4 (Rtn4) and Rab33b (84, 115). Deletion of all four of these effectors together, but not individually, impairs intracellular growth, which can be restored with complementation of just SdeA in Dictyostelium discoideum (115, 116). Interestingly, analysis of the genomes available on NCBI by BLAST shows that Rtn4 and Rab33b homologs can be found in D. discoideum, Tetrahymena thermophila, and Naegleria gruberi, but not other Tetrahymena spp., Naegaleria spp., and Hartmannella spp., indicating a possible host-specific requirement for the SidE family in protists.

Redundancy in microbes is often lost over time, particularly in obligate and facultative intracellular pathogens, but L. pneumophila has retained a large number of seemingly redundant effectors (74, 117). Growth of L. pneumophila in a variety of environmental protist hosts and temporal regulation may explain why L. pneumophila has retained these effectors, especially given that protein composition and regulatory mechanisms vary within a broad range of hosts. An arsenal of more than 320 effectors is likely what is responsible for the ability of L. pneumophila to replicate within diverse environmental hosts. The effectors likely constitute an arsenal, in which effectors represent armaments that may be specific for each protist host. L. pneumophila can use any combination of armaments in order to survive intracellularly within a certain protist host. It may seem counterintuitive, but Legionella may represent a genus of highly evolved and evolutionarily fit organisms that retain the ability to survive in a broad range of hosts and thus is the ultimate generalist pathogen.

Effector redundancy, as well as variation, is a prominent feature among Legionella spp. (113). In addition, members of the genus contain their own unique set of effectors, which vary from 52 to more than 300 putative effectors (106). Of the 41 Legionella spp. analyzed, 30 effectors were identified in 31 to 40 species, while 78% of Legionella effectors are shared by only 10 or fewer species (106). L. pneumophila contains 30 species-specific effectors (106). Interestingly, only seven effectors were identified to be present across the genus, including LLAPs (106). These seven proteins are designated as “core effectors,” although the function of most is unknown: AnkH, MavN (iron acquisition), RavC, VipF (GNAT family *N*-acetyltransferase), cetLp1, Lpg3000, and Lpg2832 are present in all 41 Legionella spp. tested (106, 118–120). Remarkably, AnkH/Lpg2300 is the only effector also found in Coxiella and Rickettsia, which both utilize a Dot/Icm T4SS (106). These core effectors likely modulate highly conserved eukaryotic process, may represent some of the most important armaments in the L. pneumophila arsenal of effectors, and may account for the broad range of protist hosts for L. pneumophila.

## EVOLUTION OF THE LARGE ARSENAL OF L. PNEUMOPHILA EFFECTORS THROUGH ACQUISITION FROM PROTIST HOSTS

Many L. pneumophila effectors contain eukaryotic protein domains and motifs such as the F-box, U-box, ankyrin repeats, SEL-1 repeats, prenylation motifs, and other posttranslational modification motifs (44, 45, 121–123). These L. pneumophila effectors are involved in modulation of a plethora of host processes, which include, but are not limited to, signaling, vesicular trafficking, apoptosis, protein synthesis, ubiquitination, histone modification, posttranslational modification, etc., aiding in their ability to interfere in host processes using eukaryotic domains (7, 44, 45, 74, 86, 122, 124). Examination of the evolution of effectors may provide some clues.

The difference between the G+C content of core effectors (37.4%) and the genome (38.3%) is minimal, suggesting both have evolved as part of the Legionella genus over an extended period of time (106). However, the G+C content of species-specific effectors (∼34%) is consistently lower than the G+C content of the genome for all tested Legionella species, indicating that these genes might have been recently acquired, after speciation (106). Thus, the majority of the effectors may have been acquired more recently. Interestingly, similar to the G+C content of L. pneumophila effectors, protist genomes are typically characterized by a low G+C content (26.4%) (125). The long-term coevolution of L. pneumophila with various protists has likely influenced the genomic content of this organism through interkingdom horizontal gene transfer (HGT) (121, 122, 126, 127).

Even within strains of the same Legionella species, a high degree of plasticity is observed (44). Between L. pneumophila strain Paris and L. pneumophila strain Lens, 2,664 genes are conserved, but 428 and 280, respectively, are strain-specific genes (44). Potential hot spots for genomic rearrangement have been identified that contribute to the plasticity of the genome (44, 128). L. pneumophila strains contain plasmids that remain independent and/or have been integrated into the genome (44).

The L. pneumophila genomic plasticity and long-term coevolution with numerous species of protists, intra-amoebal species, and amoebal endosymbionts likely has contributed to the arsenal of effectors in L. pneumophila. Genes acquired by Legionella through interkingdom HGT and other intraprotist prokaryotes, such as endosymbionts, have likely been the major sources of eukaryotic-like genes in Legionella. Many of these effectors contain eukaryotic proteins or eukaryotic-like domains and motifs (7, 44, 127). Protists may act as the gene melting pot, allowing diverse Legionella species to evolve by gene acquisition and loss and then either adapt to the intra-amoebal lifestyle or get digested as a food source.

L. pneumophila is a naturally competent organism that takes up DNA through conjugation as well as natural transformation (129–131). Evolution of host genes acquired by L. pneumophila through HGT into a translocated effector is a complex process that likely requires a long time of coevolution. Long-term convergent evolution and modification of the genes acquired through HGT involve splicing of introns, acquisition of prokaryotic promoters and regulators, evolution of Dot/Icm-dependent translocation motifs and posttranslocation modification motifs, and interaction with a Dot/Icm chaperone (126). It is to be expected that many of the eukaryotic-like proteins in L. pneumophila are still undergoing convergent evolution through modifications that might enable them to become translocated and functionally active effectors within the host cell (121).

## WHEN L. PNEUMOPHILA FAILS TO ADAPT TO THE INTRACELLULAR LIFE WITHIN A PROTIST HOST: LESSONS TO BE LEARNED

Even though L. pneumophila contains a plethora of effectors for intracellular survival within various hosts, it still cannot grow in all protists. Amaro et al. characterized three types of interactions between L. pneumophila and protists that do not result in intracellular replication of L. pneumophila: host avoidance of L. pneumophila uptake, ingestion and subsequent release of L. pneumophila in pellets, and digestion of L. pneumophila (Fig. 2) (132).

Historically, taking a pathogenic-centric view on infection, how L. pneumophila interacts with these types of restrictive protozoa is unknown. Interestingly, the group of protozoa that releases L. pneumophila without digestion represents an intermediate stage between being able to be taken up but not digested. In these hosts, the mechanism that fails to allow biogenesis of the LCV but still prevents host grazing is unknown. Presumably, L. pneumophila is still able to subvert lysosome fusion. However, the host still manages to overcome parasitosis by releasing L. pneumophila. In these organisms, there are many possibilities for why L. pneumophila fails to replicate. The failure to establish the LCV could be derived from a failure to intercept ER-derived vesicles by the LCV. L. pneumophila may fail at polar delivery of Dot/Icm effectors, preventing LCV biogenesis (111, 112). Alternatively, the host may have a unique primitive innate mechanism that L. pneumophila is not equipped to modulate.

The protist hosts that can avoid uptake of L. pneumophila could provide more detailed insight into the mechanism of attachment and phagocytosis. To complicate matters, Acanthamoeba S13WT harboring endosymbiotic Neochlamydia eS13 resists L. pneumophila infection by preventing entry (133–135). The presence of other intracellular organisms could alter the permissiveness of the protist to allow or inhibit intracellular replication of L. pneumophila. Research has barely scratched the surface of these types of multispecies interactions, which is likely due to our scant knowledge of protist biology and genetics, as well as the lack of tools to study L. pneumophila-protist interactions. Undoubtedly, these types of interactions will be difficult to identify and study but will give a realistic picture as to how the intracellular environment of the protist shapes L. pneumophila pathogenicity.

One recently identified amoeba that consumes L. pneumophila is Solumitrus palustris, a percolozoan most closely related to Allovahlkampfia spelaea, which may be able to harbor pathogenic bacteria (132, 136, 137). Legionella steelei induces “food poisoning” in S. palustris, causing the death of the host without intracellular replication, under conditions of high bacterium-to-protist ratios (132). The data shown by Amaro et al. suggest L. pneumophila is consumed by S. palustris through autophagy (132). L. pneumophila is unable to translocate Dot/Icm T4SS effectors in S. palustris at either detectable levels or at all, possibly due to failure to localize the Dot/Icm machinery to the poles (132). The mechanism(s) by which L. pneumophila fails to prevent digestion by some protists could highlight where redundancy in avoiding autophagy or preventing lysosome fusion is ineffective.

Willaertia magna represents a species of amoeba that is permissive to L. pneumophila. However, it has been shown that one strain, W. magna c2c, was capable of inhibiting the growth of L. pneumophila strain Paris but not the Philadelphia or Lens strains (138). W. magna c2c is being considered for commercial use in Europe as a bioremediation treatment against L. pneumophila in water systems (139). This finding received little attention in the field, but it should be revisited for the importance of strain-related virulence and redundancy of effectors in L. pneumophila. What effectors have been lost/gained between Paris, Lens, and Philadelphia that allow for this differential pathogenicity phenotype to a specific protist? Additionally, what host factors about W. magna c2c changed to make it resistant to the Paris strain? L. pneumophila and W. magna c2c may represent the tug-of-war between host resistances and bacterial pathogenesis and should be deciphered. Resistance to grazing by protists has likely been a strong evolutionary driver for evolution of L. pneumophila within various protists. Long-term coculture of L. pneumophila with a Legionella*-*resistant protist may allow for a gene drive toward pathogenicity in the resistant host. However, the lack of the melting pot of genes that L. pneumophila has access to in the environment could hinder this experiment. Enhancing the coculture of L. pneumophila and the Legionella*-*resistant protist with an intracellular organism(s) known to replicate in the resistant amoebae would be a better real-time experiment for pathogenic gene drive. If individual mutations are all that is required to overcome a restrictive host, advances in high-throughput screens could harness mutagenesis libraries of L. pneumophila to determine additional factors necessary for intracellular replication (140). However, this is unlikely considering the complexity for an acquired host gene to evolve and code for a translocated effector. In the same vein, high-throughput screens of L. pneumophila strains could provide for better understanding of host restriction of some strains but not others and relate that to effector contents.

Two major possibilities exist for failure of L. pneumophila to replicate within a protist host: requirement of additional effectors or requirement of further evolution of protist genes acquired though interkingdom HGT. While work has started to answer the question on the minimal genome needed for L. pneumophila to successfully replicate in mouse macrophages, one may wonder what is the largest effector arsenal L. pneumophila could have? At each step of the way, the number of effectors utilized by L. pneumophila is staggering. Would acquisition of more effectors allow for even broader host capacity or the ability to overcome restriction of a protist host? The foundation for a larger arsenal of effectors is already available, within Legionella species that harbor ∼6,000 effectors (106). What is the limiting factor on the number of unique effectors an organism can utilize? Will congestion of traffic through the Dot/Icm translocation apparatus or insufficient delivery of effectors become an issue?

## MACROPHAGES VERSUS PROTIST HOST MODELS FOR STUDYING EFFECTORS

Unfortunately, most species of protists are poorly characterized or difficult to grow in the lab, with limited tools, genomic information, or cellular and biochemical studies. This difficulty had led researchers to study L. pneumophila pathogenesis in human or mouse macrophages or D. discoideum. The ability of L. pneumophila to cause disease has likely been impacted by the fact that macrophages are similar to primitive phagocytes, protists, in their basic biology of phagocytosis and degradation of particles. Too much emphasis is placed on pathogenicity in mammalian hosts as being the prime determinant for L. pneumophila pathogenicity. The crux of intracellular replication of L. pneumophila in macrophages is its capacity to replicate within numerous protist hosts and the redundancy of effectors that constitute an arsenal to deal specifically with each host within a broad range of hosts.

While the basic biology of macrophages and that of phagocytic protists are thought to be similar enough to allow for intracellular replication of L. pneumophila, there are major notable differences between the two evolutionarily distant phagocytic host cells upon injection by L. pneumophila. In macrophages, L. pneumophila prevents host apoptosis through triggering NF-κB-dependent and -independent antiapoptosis processes to support intracellular replication (141–143), possibly to the hindrance of egress, whereas in A. castellanii, an increase in pyroptosis may facilitate bacterial egress (144–147). Macrophages have caspases, which are the executioners of apoptosis, while protists have metacaspases and paracaspases (148–150). Metacaspases that are cysteine proteases share structural similarity to caspases (148). L. pneumophila could be activating metacaspases or paracaspases in the protist host in a similar manner to caspase-3 activation in human macrophages (54, 151, 152). Unlike protists, macrophages do not graze on microbes. Rather, their innate function is to kill the invading pathogen, albeit, mechanisms to evade grazing by protists may have contributed to the protection of L. pneumophila degradation by the macrophage.

However, the ability of L. pneumophila to interact with processes that are only known to be present in higher multicellular eukaryotes, like NF-κB-dependent transcription and antiapoptotic mechanisms (141, 153), poses an interesting question regarding the evolution of L. pneumophila and the simple hypothesis that environmental aerosol transmission as a result of our own industrialization was all that was needed for transmission of L. pneumophila to reach out and infect the “accidental” human host. To date, no single-cell organism or even a simplistic animal such as Hydra, choanoflagellates, or even Caenorhabditis elegans, which L. pneumophila can infect, has been shown to have NF-κB (154, 155). It is possible that primitive NF-κB-like transcription factors may exist in primitive eukaryotes that are similar enough to allow for function in macrophages. Interaction with integrin may also highlight host evolutionary differences (156). However, it is more likely that, prior to successful infection of humans, L. pneumophila has adapted to and coevolved with multicellular environmental organisms in which the pathogen has evolved to modulate cellular processes specific to higher multicellular eukaryotes that are absent from unicellular ones.

Indeed, it has been shown that loss of one-third of L. pneumophila effectors results in a defective phenotype of L. pneumophila in protists, but not mouse macrophages (114). This is evidence for the high redundancy of effectors in L. pneumophila, since the mutant with mutation in five gene clusters encoding ∼31% effectors is still capable of intracellular replication in mouse macrophages but not D. discoideum (114). With the exception of the mouse A/J strain, all inbred mouse strains restrict L. pneumophila by Naip5 recognition of L. pneumophila flagellin and rapid host cell pyropoptosis (3, 157–160), which is evaded in human macrophages (161). Permissive A/J mouse macrophages handle L. pneumophila differently from human macrophages (157, 162–164). The lag phase of growth of L. pneumophila in A/J mouse macrophages is longer than that in human macrophages: 8 to 10 h versus 4 h (165–167). The overall trafficking of L. pneumophila in A/J mouse macrophages is different from that in human macrophages (168). Unlike human macrophages or protists, within the permissive A/J mouse macrophages, L. pneumophila resides within a vacuole that acidifies and merges with the lysosome and autophagy machinery by 16 h postinfection (162). However, D. discoideum autophagy mutants have no effect on L. pneumophila intracellular replication, and thus, this is likely a mouse-specific process (169).

Deletion of few of the more than 320 effectors causes a decrease or loss in the ability of L. pneumophila to replicate intracellularly in macrophages. There are numerous unanswered questions about the evolution of L. pneumophila to infect humans. Why have we not seen the opposite, where an effector mutant causes a more robust replication? Is it possible that the presence of some effectors that manipulate cellular processes unique to protist hosts may become like anti-virulence factors in macrophages, reducing ability of L. pneumophila to replicate? It is possible that some protist-specific effectors of L. pneumophila could backfire in the human host, as they may lead to hazardous accidental activation of innate immune responses. Is L. pneumophila really able to replicate in the human macrophage so seemingly flawless? Will anti-virulence factors be identified as an accident due to the evolution of L. pneumophila within protists? Regardless, L. pneumophila has clearly evolved with powerful mechanisms to overcome macrophage innate immunity.

Our knowledge is being limited by the use of macrophages as the sole host to determine the role of L. pneumophila effectors in intracellular replication. While D. discoideum and Acanthamoeba are very common environmental hosts of L. pneumophila, there is bias toward their sole use in determining environmental pathogenicity. Even so, V. vermiformis is more commonly identified with Legionella spp. in water systems than Acanthamoeba (22, 170, 171). Excluding the wide range of pathogenic potential by examining only one type of environmental host will provide limited knowledge. Future studies on effector testing should consist of protist host panels rather than only human or mouse macrophages, taking into consideration evolutionarily diverse hosts, like Tetrahymena, Naegleria lovaniensis, and even resistant (S. palustris) or selectively resistant (W. magna c2c and Acanthamoeba S13WT) protists. Although this approach still does not represent the remarkable diversity among protist hosts for L. pneumophila, it would be a better representation of the broad unicellular host range and the role of the arsenal of “redundant” effectors in various hosts, and many of their armaments may not be applicable to human macrophages.

## CONCLUSION

L. pneumophila may be the most generalist bacterial pathogen known. With the help of its arsenal of effectors, L. pneumophila has the capacity to infect protists of the kingdoms Protozoa and Chromista. Redundancy within the arsenal of L. pneumophila effectors likely aids in its ability to replicate intracellularly within a broad host range of unicellular eukaryotes. Limited tools exist for studying the relationship between the evolution of protist-specific cellular processes and the ability of L. pneumophila to infect human macrophages, and many of the redundant effectors may have evolved to specifically modulate unicellular eukaryotic processes that are absent in metazoans.

However, L. pneumophila can still be consumed by some protists or have intracellular replication blocked, but little is known about interactions between L. pneumophila and resistant protists. Studying the relationship with permissive and nonpermissive protist hosts would provide better understanding of effector evolution, function, and requirement for intracellular replication.

L. pneumophila modulates some cellular processes known to be present only in higher eukaryotic organisms but not unicellular protists. This could indicate that L. pneumophila may also have coevolved with multicellular eukaryotic organisms in the environment, rather than just unicellular protists. Therefore, infection of human macrophages by L. pneumophila may not have been a simple accident.
